# A dynamics-based model of multi-state rumor propagation with triple identities

**DOI:** 10.1038/s41598-025-22166-0

**Published:** 2025-11-12

**Authors:** Zhiqiang Su, Ailian Wang, Xuyang Gao, Tao Li, Yangqi Zheng

**Affiliations:** College of Computer Science and Technology (College of Data Science), Taiyuan University of Technology, Taiyuan, 030024 China

**Keywords:** Rumor spreading, Dynamic model, Social network, Differential equation, Immunology, Mathematics and computing

## Abstract

With the wide application of social networks, rumor spreading has emerged as a critical issue affecting social stability and public order. In order to deeply study the dynamic characteristics of rumor propagation, this paper proposes a dynamics-based multi-state rumor propagation model with triple identities. The model combines individual identities (rumor publishers, rumor controllers, and ordinary participants) and multiple dynamic states of individuals (e.g., susceptible, latent, and ignorant spreaders, etc.) in social networks, and describes the rumor spreading process in social networks by differential equations. With the model, rumor publishers influence ordinary participants by spreading rumors, while rumor controllers prevent the further spread of rumors through immune mechanisms. Meanwhile, the propagation status of individuals changes over time, including the transition from susceptible to ignorant propagator and malicious propagator statuses. By simulation experiments using real social network data, the effects of different network structures, propagation parameters and control strategies on rumor spreading are evaluated. The results show that individual identity and network structure have a significant impact on rumor spreading, and the effective intervention of rumor controllers can significantly slow down the speed and scope of rumor spreading. This paper provides a theoretical basis for comprehension of the rumor spreading mechanism and establishment of rumor prevention and control strategies, and offers new ideas for public crisis management based on social networks.

## Introduction

In modern society, as a typical social communication phenomenon, rumors are widely prevalent in all types of social networks and information dissemination channels. With the rapid development of information technology and the Internet, the speed and influence of rumor dissemination have also greatly increased, posing potential risks to social stability and public security. Rumors can not only rapidly change public perception and behavior, but may also trigger social panic, create misunderstanding, and even adversely affect the economic and political environment. Therefore, in- depth study of the mechanism of rumor spreading and exploration of effective control strategies have become important topics in the fields of social sciences, communication science and network science^1,2^.

Research on rumor models began in the 1960s^3^. Most of the existing rumor spreading models are based on epidemic models as rumor spreading shows interesting similarities with epidemic spreading^4,5^. Daley and Kendall^3^ first proposed a basic DK model for rumor spreading. Maki and Thomson^6^ focused on analyzing rumor spreading models based on mathematical theories and developed the MK model. The DK and MK models have been widely used in quantitative studies of rumor propagation^7–13^, but the major drawback of these models is that they do not take into account the topological features of social networks and are not suitable for describing the rumor propagation mechanism on large-scale social networks. Therefore, scholars have gradually taken into account the effect of network topology on rumor spreading^14–17^. Focusing mostly on small-world networks and scale-free networks, Watts and Strogatz^18^ presented the concept of small-world networks to describe the transition process from completely regular networks to completely random networks. While Barabási et al.^19^ first presented the Barabási-Albert (BA) network, a special case of scale-free networks, which exhibits a power-law degree distribution, which means that only a few nodes in a scale-free network have very high degrees while most of the nodes have relatively lower degrees, similar to many of the large and complex networks in the real world, and the results show that scale-free networks are often considered preferentially^20,21^.

Cheng, Zhu and other scholars^22–25^ systematically studied the influence of psychological variables such as anxiety, attitude tendency and cognitive deviation on communication, and developed targeted intervention strategies. Chen et al^26,27^ improved the accuracy of communication prediction by constructing a differential equation model including information credibility and audience relevance; Wang et al.^28^ found that the heterogeneity of node recognition ability will significantly affect the scale and scope of rumor outbreak. Dong^29^ focuses on emotional dynamics and reveals the interaction mechanism between emotional state and rumor-dispelling behavior. In addition, the academic community has also built a model from the dimensions of hesitation mechanism^30^ and age structure^31^, and found that the people who spread rumors are gradually changing.

Huo^32^ proposed an I2SR rumor spreading model, which takes into account the activity of the nodes and divides the spreaders into high active spreading rate and low active spreading rate. Both Liu^33^ and Xia^34^ proposed SEIR rumor propagation models with considering hesitation and obtained that the ambiguity of rumors can effectively reduce the impact of rumor propagation. In addition, Liu^33^ discussed immune strategies for rumor propagation and Xia^34^ concluded that scale-free networks are faster and more truthful than small-world networks. Chen^35^ proposed the influence of rumor credibility. Wang^36^ proposed a social network information dissemination model based on the relative weight of users. But none of them considered the influence of real-world identities. Dong^37^ mainly investigates a rumor propagation model with dual identities, exploring the dynamic mechanisms of rumor spreading in complex networks and its control strategies. Hu^38^ studies how different individuals’ attitudes towards rumors influence the diffusion process of rumors.

Inspired by the previous research work, we realized that the rumor propagation dynamics model they constructed mainly focuses on designing from the macro level of the network, however, some important details are still missing in the model, which are the issues that our model aims to address, as reflected in the followings:

- Traditional communication models tend to assume that all individuals behave in the same way during communication, ignoring the differences between individuals. Our model takes into account the different roles and behaviors of individuals in the propagation process by introducing their identities (e.g., rumor publisher, rumor controller, and ordinary participant). Each identity corresponds to different dissemination capabilities, dissemination intentions, and reactions to rumors. For example, a rumor publisher may actively spread the rumor, while a rumor controller may try to curb the spread of the rumor, and an ordinary participant plays a more passive role in the dissemination. By such identity segmentation, our model could more accurately reflect the communication behaviors of individuals with different roles.
- Traditional models usually focus only on the direct communication relationship between individuals, ignoring multi-level communication mechanisms such as group behavior, social opinion and media influence. Our model introduces a richer level of social interaction in the communication process, taking into account the interactions between individuals, groups and large-scale social opinion. For example, rumor publishers may influence large-scale groups through, for example, social media, while rumor controllers may intervene at the group level. Such multi-level communication effects can reflect the rumor spreading process in reality more comprehensively.
- Traditional transmission models usually use simple single states (e.g., ”susceptible” and ”infected”) to describe the behavior of individuals, while our model uses a multi-state system (including ”susceptible”, ”ignorant”, ”malicious”, ”immune”, etc.), which makes the transmission process more detailed and dynamic. For example, individuals may experience multiple state transitions during rumor propagation, and individuals in different states have different propagation behaviors and propagation abilities. Such multi-state design could more comprehensively reflect the complex changes of individuals in the rumor spreading process in reality.

In summary, this paper proposes a dynamics-based triple-identity multi-state rumor propagation model (SSEIIFR rumor propagation model), which is able to more realistically and comprehensively simulate the whole process of rumor propagation by introducing more individual identity roles and state transition mechanisms. The rest of the paper is organized as follows. The model and its differential dynamics equations are described in Sect. "SSEIIFR rumor propagation model". In Sect. "Experimental results and analysis", we conduct simulation experiments on the model based on different network structures and analyze its dynamic features by visualizing the model. Finally, we conclude the paper in Sect. “Conclusion”.

## SSEIIFR rumor propagation model

**Fig. 1:**
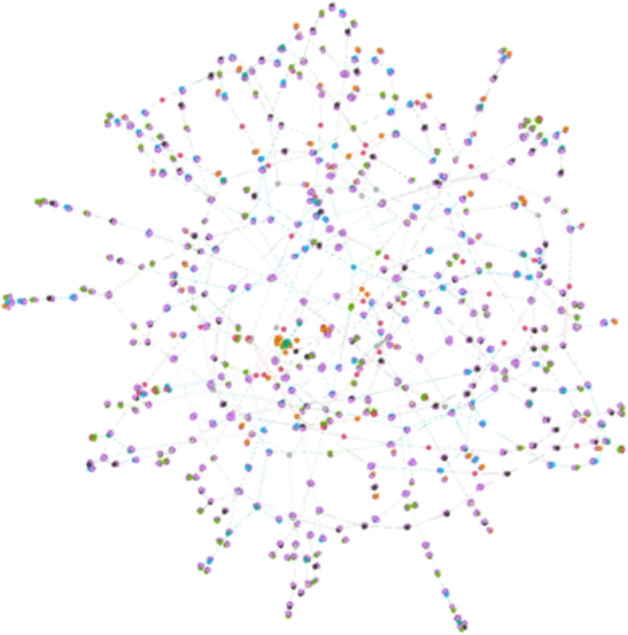
An undirected graph composed of real data set tvshow.

The model proposed in this paper is designed to describe the complex spread of information or rumors, and the core idea is introducing the interaction of multiple identities and multiple states in social networks. This type of model is commonly used in the fields of social network analysis, public health research, and online communication research, and is able to capture the interactions between different groups and their impact on the communication process more precisely. The model is based on a social network, and we define a closed and uniformly mixed group of N individuals as a social network, where individuals are vertices and person-to-person connections are edges. Then, an undirected graph G = (V, E) is obtained, where V is the set of vertices and E is the set of edges. For model simulation, we use a real dataset tvshow[?], and Fig. 1 illustrates the structure of the undirected graph composed by this dataset.

### Description of identity status classification

In social networks, each individual (node) is assigned an identity label that reflects its role or function of the individual (node) in the network. The identity of an individual determines their primary role in the communication process and influences their behaviors and the way they interact with others, therefore, these identity labels have a significant impact on an individual’s communication behavior. We assume that the identity of each node is divided into three categories: rumor publishers, rumor controllers, and general participants, which are denoted as P=P1, P2,..., Pn, C=C1, C2,..., Cm and O=O1, O2,..., Ol are denoted.

- Rumor Publishers (P): these individuals actively spread rumors, possibly for personal interests or malicious purposes. The main role of rumor publishers is to spread rumors from one node to another through social networks.
- Rumor Controllers (C): These are individuals who inhibit or prevent the spread of rumors by countering or debunking them. Rumor controllers work as a ”firewall” by persuading or otherwise immunizing neighboring nodes from spreading rumors.
- Ordinary Participants (O): These individuals are passive in the propagation process, they may be exposed to rumors or passively spread rumors due to social connections.

Each individual not only has an identity, but is also in a specific state of propagation, which describes the current state or health of the individual during the propagation process. State is a dynamic variable that changes over time based on the propagation mechanism, and an individual’s state determines whether or not they will spread rumors and whether or not they are susceptible to rumors. We designed the following individual states, which reflect the different responses of individuals to rumor propagation:

- Active person (Si): node with the ability to receive and disseminate information and is highly active.
- Inactive person (Sa): node that has the ability to receive and spread information and is not highly active.
- Infiltrator (E): node that has been exposed to the rumors but has not yet started spreading them.
- Ignorant spreader ($I\_m$): node that spreads rumors unconsciously.
- Malicious disseminator ($I\_n$): node that consciously spreads false information.
- Wanderer (F): node that is judging whether the information is false or not but is still spreading false information.
- Immune (R): node that is already immune, i.e. no longer involved in spreading rumors.

**Fig. 2:**
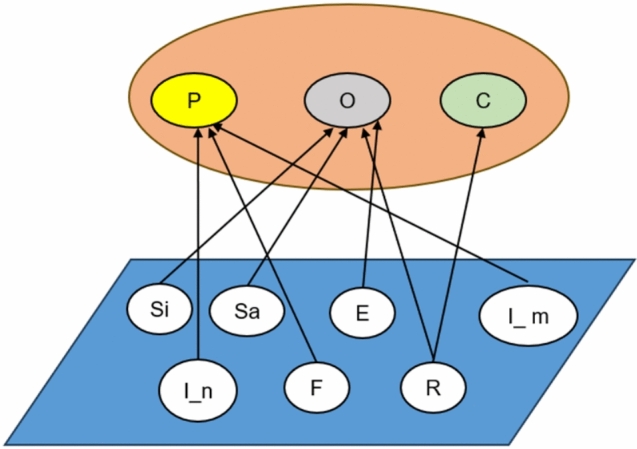
Structure of correspondence between real identity and state.

There exists a mutual correspondence between the real-world identity and the various states of propagation, and the specific relationship is shown in Fig. 2. The upper layer is the real-world identity, and the lower layer is the various states in the propagation process.

In the model, rumor publishers include malicious spreaders, ignorant spreaders, and wanderers, while rumor controllers consist of immunizers, and ordinary participants include active, inactive, infiltrator and immunizers. Based on the initial conditions of the model, all rumor publishers are assumed to be malicious spreaders, i.e., these individuals are initially active in spreading false information; all rumor controllers are immune, who are no longer affected by the rumors and are able to prevent other users from being misled by the rumors; and for ordinary participants, all of them are set to be susceptible in the initial state, where susceptible participants refer to those who have not yet come into contact with the rumor controller or rumor publisher, and may be active or inactive.

With the spread of information in social networks, ordinary participants interact with other individuals and thus experience the transformation of their identities and communication status. Specifically, when ordinary participants encounter rumor controllers, they are affected by disinformation, rebuttal, or other immune mechanisms and gradually become immune, i.e., they are no longer affected by rumors and do not actively spread false information. However, when ordinary participants encountered rumor publishers, they were easily misled or influenced by them, and then transformed into ignorant spreaders. Although these individuals do not realize that the information they spread is false, they unconsciously pass on the rumor to other nodes in their social network, further expanding the spread of the rumor. The presence of ignorant spreaders is usually one of the main reasons for the spread of rumors, as they act without malicious intent resulting in the widespread dissemination of false information. As the spreading process progresses, some ordinary participants are eventually transformed into immunizers. This transformation process is partly achieved through initial contact with rumor controllers, while the majority of them become more alert and eventually immune to the influence of rumors as they continuously come into contact with, learn about, and understand the relevant knowledge in the process of rumor spreading. They will become a new group of immune people in the social network, capable of identifying and counteracting the spread of false information, thus forming a kind of ”anti-rumor” protective barrier.

### Establishment of differential equations

In order to accurately describe the spreading process of rumors in social networks, we develop a set of differential equations based on SEIR model. The state of each individual changes over time and is interdependent with the propagation behavior of its neighbors. Specifically, the transitions between states are described by a series of differential equations as follows:

Si was infected with infection rate $\beta$ after contact with $I\_m$.

1 $$\begin{aligned} \frac{dS_i}{dt} = -\beta \frac{S_i}{N} I\_m \end{aligned}$$

Sa was infected with infection rate $\varphi$ after contact with $I\_m$.

2 $$\begin{aligned} \frac{dS_a}{dt} = -\varphi \frac{S_a}{N} I\_{m} \end{aligned}$$

E comes from Si and Sa, and is converted into $I\_m$ with probability $\sigma$ and R with probability $\theta$.

3 $$\begin{aligned} \frac{dE}{dt} = \beta \frac{S_i}{N} I\_{m} + \varphi \frac{S_a}{N} I\_{m} - \sigma E - \theta E \end{aligned}$$

$I\_m$ comes from E, and is converted into In with probability $\alpha$ and R with probability $\eta$.

4 $$\begin{aligned} \frac{dI\_m}{dt} = \sigma E - \gamma I\_{m} - \alpha I\_{m} \end{aligned}$$

$I\_n$ comes from $I\_m$, and is converted into R with probability $\eta$

5 $$\begin{aligned} \frac{dI\_n}{dt} = \alpha I\_{m} - \eta I\_n \end{aligned}$$

F comes from $I\_m$, and is converted into R with probability $\lambda$

6 $$\begin{aligned} \frac{dF}{dt} = \gamma I\_{m} - \lambda F \end{aligned}$$

R comes from F, $I\_n$ and E

7 $$\begin{aligned} \frac{dR}{dt} = \lambda F + \theta E + \eta I\_n \end{aligned}$$

P is the growth of all $I\_m$, $I\_n$ and F.

8 $$\begin{aligned} \frac{dP}{dt} = \frac{dI\_m}{dt} + \frac{dI\_n}{dt} + \frac{dF}{dt} \end{aligned}$$

C is constant, and its change rate is 0.

9 $$\begin{aligned} \frac{dC}{dt} = 0 \end{aligned}$$

O refer to the total number of users except C and P.

10 $$\begin{aligned} \frac{dO}{dt} = \frac{dSi}{dt} + \frac{dSa}{dt} + \frac{dE}{dt} + \frac{dR}{dt} \end{aligned}$$

In this model, we use real social network datasets to build a network topology in which we establish the following rules:

**Rule 1**: Given that users in state Si are very active on the Internet and receive information faster, they will transform to state E with a high probability after receiving a rumor, we assume that users in state Si will transform to E with a probability of $\beta$ after being exposed to state $I_m$ or state $I_n$.

**Rule 2**: As users in state Sa are not active on the Internet and there is a delay in receiving information, users in state Sa are then transformed to state E with a smaller probability $\varphi$.

**Rule 3**: When users are in state E, based on their education level and various external factors, these users will be divided into two categories: those who can identify the rumor on their own and those who are less capable of distinguishing the rumor. The former can clearly identify the rumor and transform to state R with $\theta$ probability, while the latter will transform to state $I_m$ with $\sigma$ probability and continue to spread the rumor.

**Rule 4**: During rumor propagation, most of the users in state $I_m$ spread the rumor without knowing the truth of the rumor, but a small fraction of them continue to spread the rumor even after they have learned the truth of the rumor, with probability $\alpha$, and ultimately, this fraction will be transformed into state $I_n$. And most of the users will be in a state of validation after they have learned the truth of the rumor, which we call the Wanderer F, and the conversion into F with probability $\gamma$.

**Rule 5**: As time goes by, users in state F come to their senses and confirm those rumors and transform them into R with probability $\lambda$.

**Rule 6**: In the end, those malicious propagators $I_n$ are punished by the law, arrested with probability $\eta$, and convert to R after being punished.

**Fig. 3:**
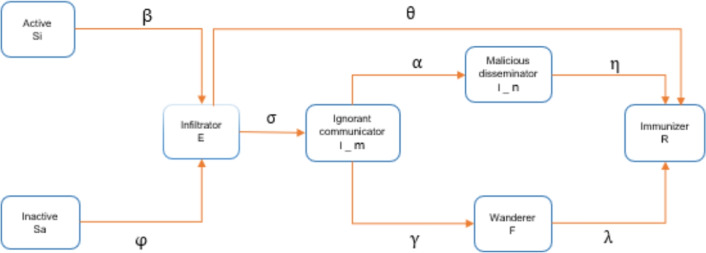
Flowchart of rumor spreading process.

In order to demonstrate the transformation process more intuitively between the various states, we construct a flowchart of the rumor propagation process, as shown in Fig. 3. The meaning of the parameters of the model is summarized in Table 1.

**Table 1:** model parameters.

Parameter	Parameter description	Parameters	Parameter description
$\beta$	Probability of being infected by $S_i$.	$\varphi$	Probability of being infected by $S_a$.
$\sigma$	Probability of being infected by *E*.	$\theta$	Probability of identifying rumors by *E*.
$\gamma$	Probability of $I_m$ recognizing the truth of rumors.	$\alpha$	Probability of malicious transmission of $I_m$.
$\eta$	Probability of being arrested by $I_n$.	$\lambda$	Probability of sobriety of *F*.

### Derivation of rumor propagation threshold

In order to systematically analyze the spreading ability of rumors in social networks, this paper deduces the spreading threshold of rumor spreading model. The basic reproduction number is an important index to measure the average number of new communicators that a single communicator can cause in a completely susceptible population during its communication cycle. It reflects whether information dissemination has persistence and diffusion potential, and is the key theoretical basis for judging whether rumors will spread on a large scale.

In this model, the propagation path can be simplified as the following structure: Ignorant spreader ($I_m$) $\rightarrow$ Infiltrator (*E*) $\rightarrow$ Become $I_m$ again. Among them, the infiltrator comes from the infection of susceptible individuals $S_i$ and $S_a$ after contact with $I_m$, and then it is transformed into $I_m$ with a certain probability $\sigma$, or it is immunized with a probability $\theta$ to quit the transmission.

By adopting the Next Generation Matrix Method, we need to construct:

F: represents the ability of each transmission variable to produce new infections;

V: represents the natural consumption (transfer-out) rate of propagation variables.

Assuming at the early stage of rumor spreading that the majority of the population is susceptible, we approximate $S_i \approx S_i^0$, $S_a \approx S_a^0$. The matrices are:

11 $$\begin{aligned} \textbf{F} = \begin{bmatrix} 0 & \dfrac{\beta S_i^0 + \varphi S_a^0}{N} \\ 0 & 0 \end{bmatrix} \quad , \quad \textbf{V} = \begin{bmatrix} \sigma + \theta & 0 \\ - \sigma & \alpha + \gamma \end{bmatrix} \end{aligned}$$

The next generation matrix $\textbf{K}$ is then computed as:

12 $$\begin{aligned} \textbf{K} = \textbf{F} \textbf{V}^{-1} = \begin{bmatrix} \frac{(\beta S_i^0 + \varphi S_a^0) \sigma }{N (\sigma + \theta )(\alpha + \gamma )} & * \\ 0 & 0 \end{bmatrix}. \end{aligned}$$

Since $\textbf{K}$ is upper-triangular, its largest eigenvalue is the diagonal principal element, therefore:

13 $$\begin{aligned} R_0 = \frac{(\beta S_i^0 + \varphi S_a^0) \sigma }{N (\sigma + \theta )(\alpha + \gamma )}. \end{aligned}$$

Where:

The molecular part: indicates the ability of communicators to trigger the infiltrator in unit time;

Denominator part: represents the average ”exit speed” of the infiltrator and spreaders;

Whole: describe the ”magnification” of the propagation chain.

If the initial susceptible population satisfies $S_i^0 + S_a^0 \approx N$, the expression simplifies to:

14 $$\begin{aligned} R_0 \approx \frac{\sigma (\beta + \varphi )}{(\sigma + \theta )(\alpha + \gamma )}. \end{aligned}$$

If $R_0> 1$: Each communicator can trigger more than one person on average, and rumors will spread on a large scale;

If $R_0 < 1$: Communication cannot be maintained, rumors will gradually disappear;

If $R_0 = 1$: The system is in a critical state, and the propagation and extinction are evenly matched.

The basic reproduction number $R_0$ defines the threshold of rumor propagation in theory, and reflects the comprehensive effects of multiple propagation paths, propagation rates and transformation probabilities. Compared with relying solely on numerical simulation, $R_0$ provides a deeper understanding of rumor spreading mechanism, and also provides a theoretical basis for subsequent communication control and intervention.

**Fig. 4:**
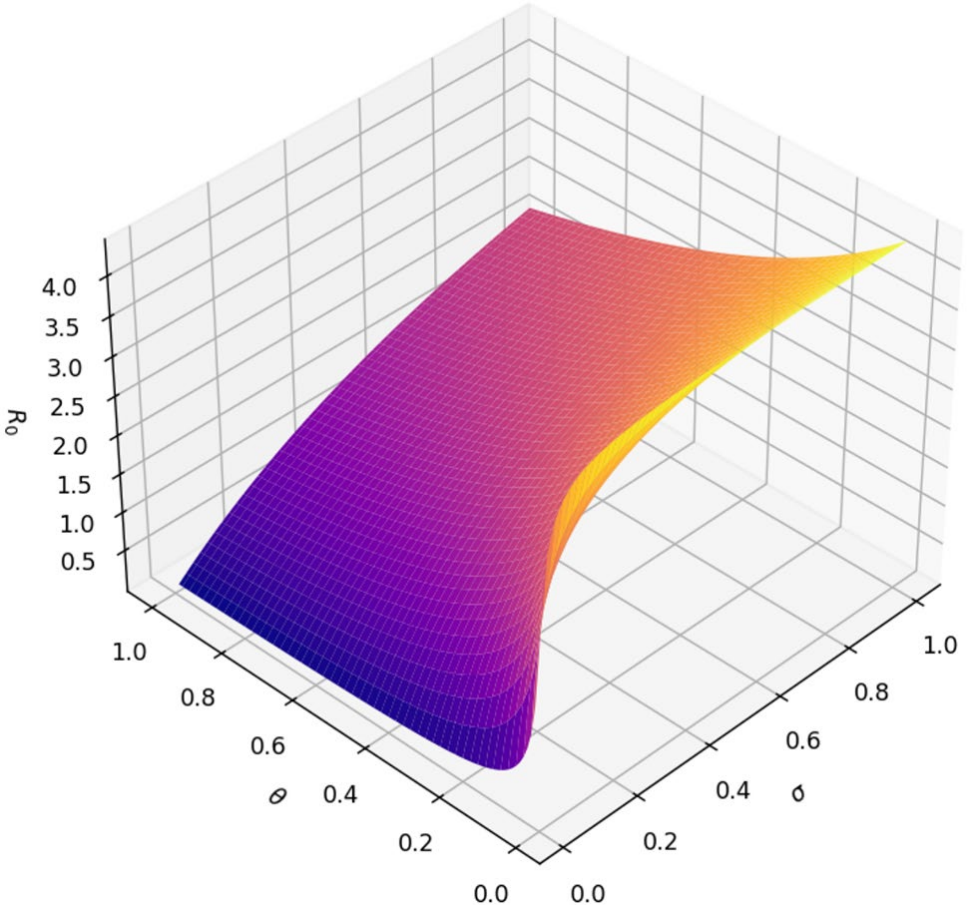
Sensitivity analysis of $R_0$ varying with conversion $\sigma$ and $\theta$.

The basic reproduction number $R_0$ is an important index to measure the dynamics of rumor spreading, which indicates the average number of secondary spreading that a single initial propagator can trigger in a completely susceptible group. In order to show the trend of $R_0$ changing with related parameters more intuitively, we draw Fig. 4, which intuitively reflects the potential risk of rumor spreading under different conditions.

Figure 4 presents the effect of variations in the exposure-to-infectious rate $\sigma$ and the exposure-to-removed rate $\theta$ on the value of $R_0$, with fixed transmission parameters $\beta = 0.9$, $\varphi = 0.8$. The figure shows that increasing $\sigma$ (faster transition from exposure to active rumor-spreading) leads to a rapid increase in $R_0$, while increasing $\theta$ (faster removal without spreading) has the opposite effect. This highlights the competing effects of activation versus suppression mechanisms in rumor dynamics. Controlling these transition probabilities can effectively shift the system below the critical threshold and suppress rumor propagation.

## Experimental results and analysis

In Section "SSEIIFR rumor propagation model", we successfully constructed an effective three-identity and multi-state rumor propagation model by establishing differential dynamics equations. In this section, we will use the model to explore the rumor propagation process in different complex networks. We will verify the scientific and rationality of the model in rumor propagation by numerical simulation.

In this paper, five real datasets are used to simulate the rumor propagation process, namely tvshow, power, PGP, food and hamsterster. All of them are sourced from the Network Repository maintained by Rossi and Ahmed^39^, as shown in Table 2. In order to systematically analyze the influence of network topology, we further constructed three types of synthetic networks, The specific parameter configuration is shown in Table 3.It is assumed that the initial time P(0)=10 is the number of initial rumor publishers, C(0)=1 is the number of initial rumor controllers, and the rest of the nodes are ordinary participants O. The state propagation among the nodes is described following alg1.

The time complexity of the algorithm is $O(n + m)$, where n is the number of nodes and m is the number of edges. The algorithm traverses each node and checks its neighbor state. Because each node and each edge are only processed for a constant number of times, the total running time is linear with the scale of the graph. For sparse graph (m $\approx$ n), the complexity is close to *O*(*n*); For dense graph ($m \approx n^2$), it is close to $O(n^2)$. This design ensures that the algorithm can run efficiently in networks of different sizes.

**Algorithm 1:**
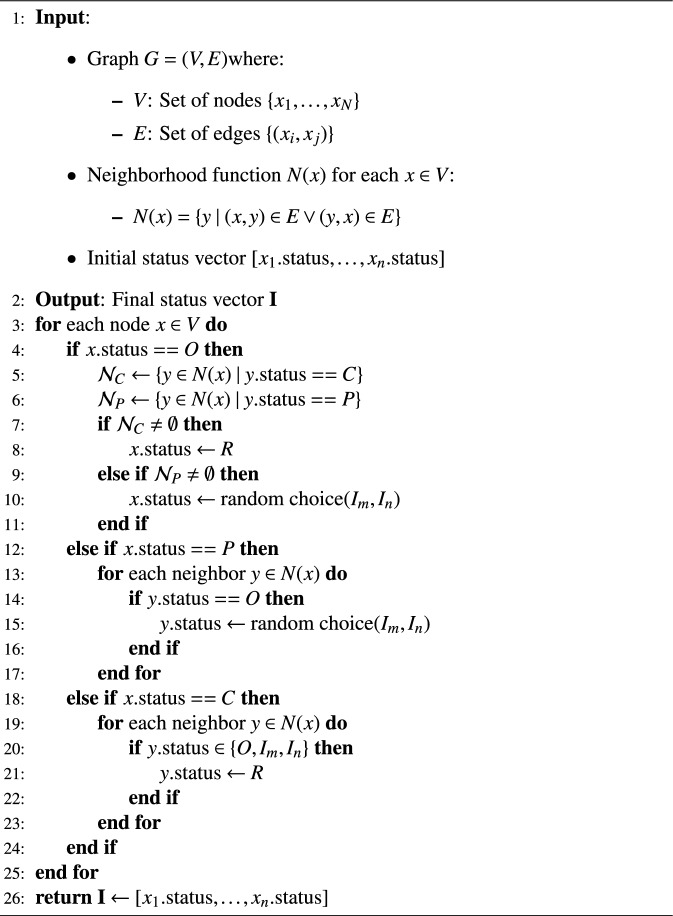
Node-Based Rumor Propagation

**Table 2:** Real data set.

Data set	Nodes	Edges	Average degree	Density
tvshow	3.9k	17.2k	8	0.00227671
power	4.9k	6.6k	2	0.000540303
PGP	10.7k	24.3k	4	0.000426403
food	0.62k	2.1k	6	0.0108969
hamsterster	2.4k	16.6k	13	0.00565353

**Table 3:** Synthetic network data set.

Data set	Nodes	Edges	Average degree	Density
LFR1	1000	997	1.99	0.001996
LFR2	5000	4998	2	0.000400
LFR3	10000	9998	2	0.000200

**Fig. 5:**
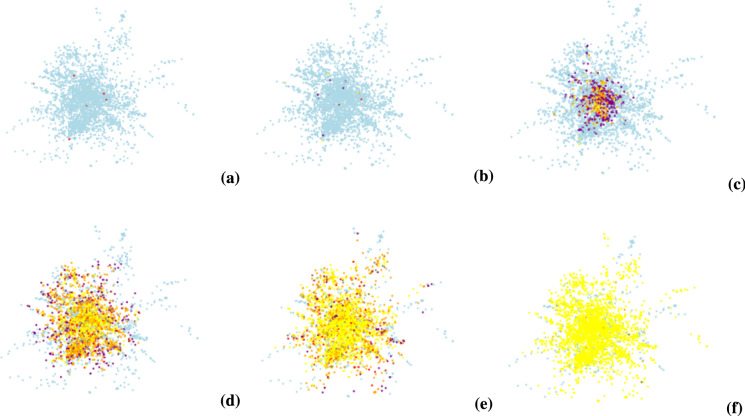
Simulation of the spreading process of rumors on real data sets.

In the early stages of the simulation, rumor publishers spread rumors to ordinary participants in their neighborhood by means of their social network connections. Based on the propagation rules, ordinary participants are infected by the rumor and gradually transform into infiltrators. Increasing numbers of incubators mean that these individuals are in the latent phase of infection and have not yet demonstrated transmission behavior, but have the ability to transform into ignorant or malicious transmitters under certain conditions. Over time, a portion of the infiltrators will transform into ignorant spreaders. Ignorant propagators begin to actively spread rumors, further influencing more individuals in the network and leading to a significant increase in their numbers. At the same time, there is a gradual increase in the number of malicious propagators in the network, individuals who not only spread rumors but may also add other disruptive elements to the propagation process. Increasing number of malicious propagators marks a peak in the influence of the rumor. At a later stage of the propagation process, a portion of individuals are transformed into immune individuals due to external factors in the network (e.g., intervention of the rumor controller, formation of herd immunity, etc.). Rumor controller C plays an important role in this process, slowing down the spread of the rumor by contacting ignorant spreaders and converting them into immunizers. As more individuals become immune, the spread of the rumor gradually narrows and slows down. Ultimately, although some of the susceptible individuals remained in their original state because they were not exposed to the rumor, the majority of individuals went through the rumor spreading process and eventually transformed into immune individuals. The rumor spreading process on this dataset is shown in Figs. 5(a) - (f).

As we can see from the propagation process of the model, the process of rumor propagation shows a typical dynamic evolution: from the initial outbreak of propagation, to the widespread dissemination in the middle stage, to the gradual fading and the formation of herd immunity in the later stage. This process is not only influenced by individual behavior, but also constrained by factors such as rumor controllers and network structure.

This process demonstrates the trend of the number of individuals with different identities and statuses over time in the rumor spreading process, and it can be clearly seen that the number of ignorant and malicious spreaders reaches a peak in a certain time period, and then due to the increase of immunizers and the intervention of the controllers, the spreading gradually tends to level off, and finally the system tends to be in equilibrium. By analyzing the experimental results, we are able to further understand the effects of different network structures and control strategies on rumor propagation, thus providing valuable references for rumor prevention and control in practice.

**Fig. 6:**
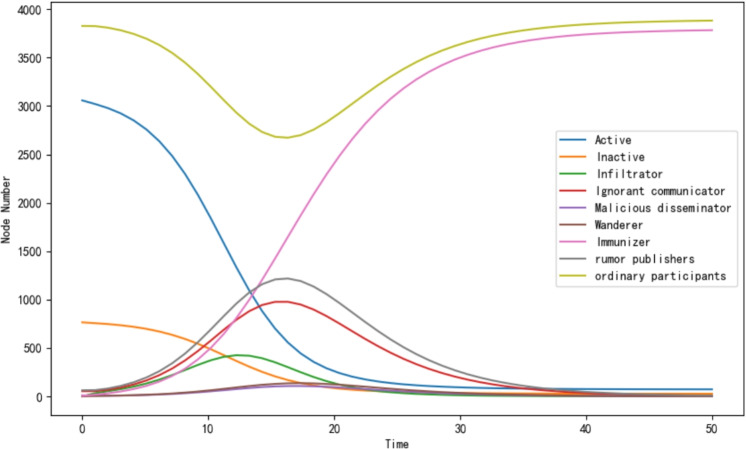
SSEIIFR rumor propagation model on tvshow.

During the process of simulation for rumor spreading model, the proportions of individuals with different identities and statuses in the social network change dynamically with the time. Figure 6 visualizes the curves of the proportion of different types of individuals as time changes in the rumor propagation process.

Different identities in the network include active person, inactive person, the infiltrator, ignorant communicator, malicious communicator, wanderer, immunizer, rumor publisher, and ordinary participant, and the parameters are set as $\beta$=0.9, $\varphi$=0.8, $\theta$=0.1, $\sigma$=0.6, $\alpha$=0.1, $\gamma$=0.1, $\lambda$=0.7, and $\eta$=0.9.

It can be seen in the figure that the number of infiltrators and ignorant spreaders rises rapidly over time, marking the initial outbreak of rumor spreading; subsequently, with the intervention of the rumor controllers and increasing proportion of the immune, spreading process is gradually suppressed, and the number of malicious spreaders and wanderers begins to decline, and eventually most of the individuals are transformed into immune, and rumor spreading is gradually calmed down.

The figure reveals the dynamic changes of different identity nodes and the herd immunity effect in the rumor spreading process. It is worth noting that as the number of rumor controllers remains constant at 1 throughout the simulation, the curve of rumor controllers is not shown in the figure in order to show the changes of other identities and states more intuitively.

**Fig. 7:**
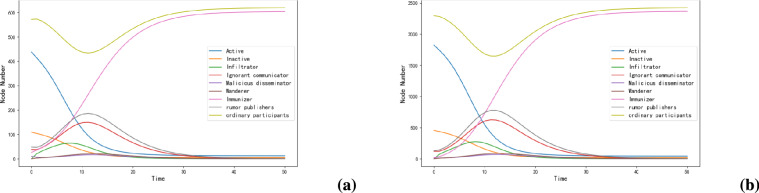
Rumor propagation model on real data set.

**Fig. 8:**
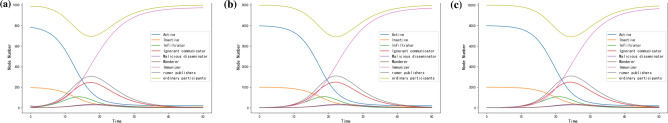
Rumor propagation model on synthetic network data set.

The experimental results show that the model proposed in this paper shows good stability in various real networks and synthetic network environments. As shown in Figs. 7 and 8, this stability feature makes the model suitable for different types of network environments and provides a reliable theoretical basis for practical application.

**Fig. 9:**
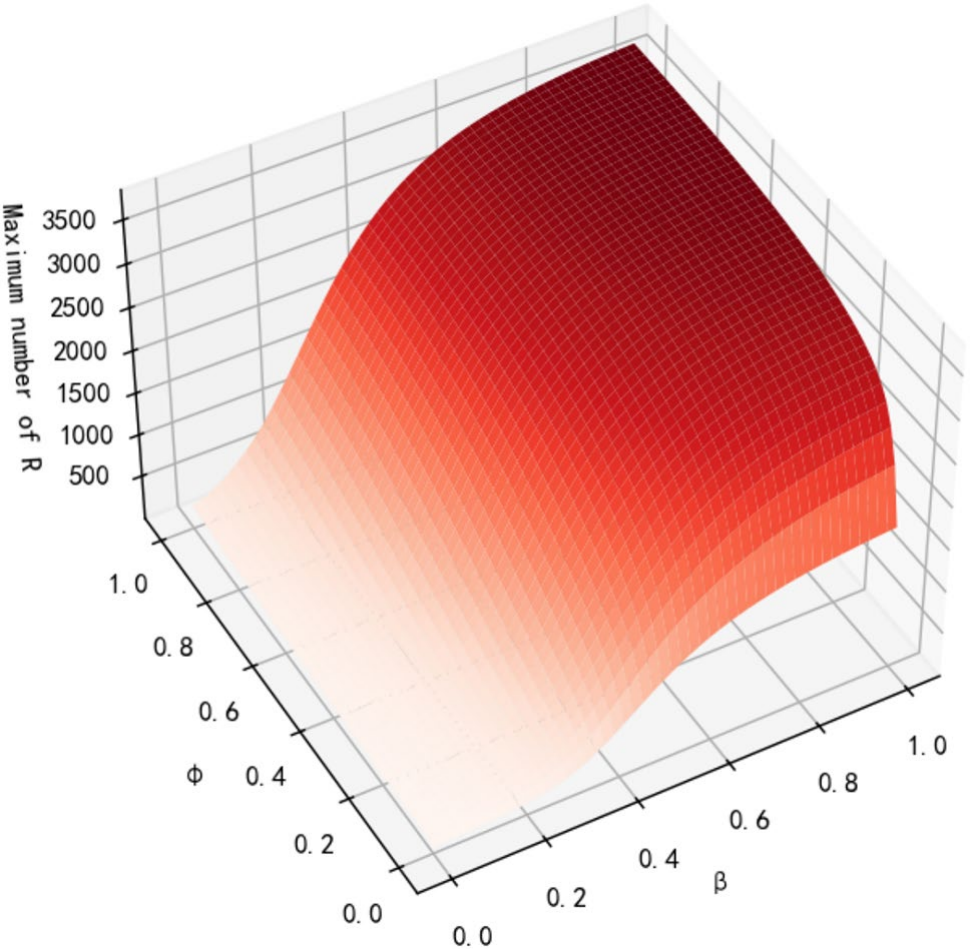
Number of Maximum R under Different $\beta$ and $\varphi$.

In this section, we explore the combined impact of the probability of being infected for vulnerable individuals (active and inactive) on rumor spreading to further understand the rumor spreading mechanism in our model. The relationship between the maximum number of individuals of class R and the two parameters $\beta$ and $\varphi$ is shown in Fig. 9, where we set 0.0 $\le$ $\beta$ $\le$ 1.0 and 0.0 $\le$ $\varphi$ $\le$ 1.0. When $\varphi$ is unchanged, the maximum number of R increases with $\beta$. When $\beta$ is unchanged, the maximum number of R increases gradually with $\varphi$, and finally converges to a stable value. When the values of both parameters are 1.0, the maximum number of R reaches the maximum value, but this is not realistic, so we choose $\beta$ = 0.9 and $\varphi$ = 0.8. When the values of $\beta$ and $\varphi$ are greater than 0.6, the curve rises slowly, and when $\beta$ and $\varphi$ are less than 0.6, the curve falls rapidly. Therefore, we can conclude that the maximum amount of R is more sensitive to $\beta$ and $\varphi$ when $\beta$ and $\varphi$ are less than 0.6.

**Fig. 10:**
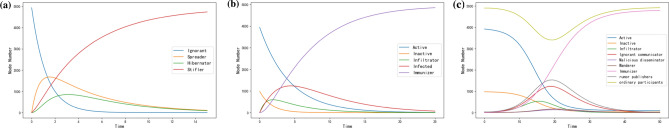
Different rumor propagation model on power.

**Fig. 11:**
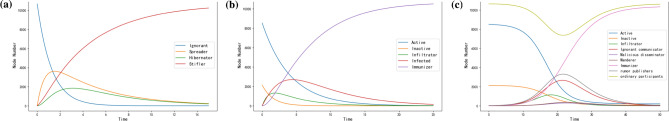
Different rumor propagation model on PGP.

In addition, we select two real networks to compare the rumor propagation models, and reproduce the SIHR rumor propagation model and RWSSEIR rumor propagation model based on the relative weight of users on these two data sets, as shown in Figs. 10 and 11. As can be seen from Figs. 10(a) and 11(a), the SIHR model has no obvious change under different data sets. Similarly, through Figs. 10(b) and 11(b), it can be seen that although the RWSSEIR model has increased to five states, the propagation speed and trend have not changed obviously. But through the model simulation on Figs. 10(c) and 11(c), we can find significant differences in propagation speed and influence range, which fully verifies the effectiveness and practicability of our proposed model.

Through comparative analysis, we can find that our model not only divides users’ identities in the real world more carefully, but also significantly improves the accuracy of rumor spreading simulation. Specifically, in terms of propagation speed, our model is closer to the real social network observation data, which is mainly reflected in the fact that the rising slope of the propagation curve is more consistent with the actual data points, and the time when the propagation peak appears is more accurate. These improvements enable the model to better reflect the temporal and spatial characteristics of information diffusion in the real world, and provide a more reliable analysis tool for studying the rumor propagation mechanism of social networks.

The experiments on these two datasets show significant differences in the speed of spreading and the scope of influence. In comparison to Fig. 6 above, Figs. 10(c) and 11(c) have slower spreading rates, which indicates that these networks have a more decentralized structure, or a smaller number of spreaders in the initial case. In contrast, in the network shown in Fig. 6, rumors may spread rapidly in a much shorter period of time due to a more tightly knit structure of the network, or a higher number of nodes affected by malicious propagators in the initial case, etc., which indicate that rumors propagation in this network is highly connected and spreads quickly.

In summary, although the specifics of each dataset (e.g., density of network connections, number of nodes, etc.) vary, the general trend across all the graphs is similar: susceptible spreaders are gradually replaced by immune spreaders over time, the number of ignorant and malicious spreaders increases and then levels off, and the system eventually converges to an equilibrium state. This similarity reveals that the mechanisms of propagation control (e.g., the interactions between rumor publishers, rumor controllers, and ordinary participants) lead to similar propagation patterns to a certain extent, regardless of the changes in the structure of the network. In other words, although the initial conditions and structure of the network may be different, under similar propagation conditions (e.g., the presence of rumor controllers, immunity rates, etc.), the system eventually reaches a similar steady state.

**Fig. 12:**
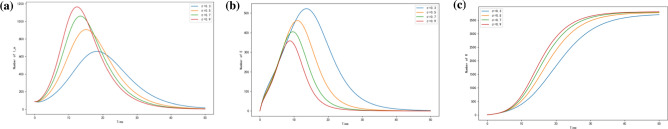
Numerical variation of $I_m$, E and R with different $\sigma$ values on tvshow.

Figure 12 show the variation of the number of E and $I_m$ in the tvshow network with $\sigma$.

As shown in Fig. 12(a), when $\sigma$ is high, it means that infiltrators are converted to infected faster, and therefore, infiltrators are rapidly converted to ignorant spreaders in a short period of time, which makes the rumor spread faster. The curve corresponding to a high $\sigma$ value in the graph will rise rapidly in a short period of time, showing a sharp increase in the number of ignorant propagators, and at the same time, due to the faster rate of conversion, a higher peak of propagation may occur, indicating that the number of ignorant propagators reaches a higher number in a short period of time. A lower $\sigma$, on the other hand, indicates a slower conversion of infiltrators into infected individuals. Therefore, although infiltrators will still be converted into ignorant spreaders, as the conversion process is slower, the rumor spread will be slower and the curve in the graph will rise more gently. On the contrary, as shown in Fig. 12(b), when $\sigma$ is high, the peak of E will be relatively low due to the faster conversion of infiltrators into infected spreaders Summarizing above, we can conclude that the value of $\sigma$ is closely related to the number of E and $I_m$ as well as the speed of rumor spreading.

Figure 12(c), when $\sigma$ is high, it means that latent individuals are converted into active spreaders faster, thus accelerating the process of rumor spreading throughout the network. Although these individuals spread the rumor faster, but at the same time, the number of immune individuals increases rapidly as they are exposed to the rumor in a shorter period of time and thus become immune. A lower $\sigma$, on the other hand, indicates that latent individuals are converted into rumor spreaders at a slower rate, resulting in a slower spreading process and a relatively slower accumulation of the number of immunizers. This shows that lower rates of conversion may lead to a slower spread of rumors, which in turn affects the formation of immunizers.

**Fig. 13:**
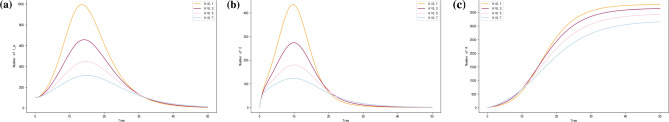
Numerical variation of $I_m$, E and R with different $\theta$ values on tvshow.

Figure 13 shows the variation of the number of $I_m$, E and R with $\theta$ in the tvshow network. As can be seen from Fig. 13(a), a higher $\theta$ value urges more The Infiltrator to be quickly transformed into immunizers, thus effectively inhibiting the spread of rumors and making the number of The Infiltrator grow more smoothly. This mechanism also limits The Infiltrator’s transformation into a communicator, resulting in a significant decrease in the number of ignorant spreaders in Fig. 13(b).

It is worth noting that Fig. 13(c) reveals an interesting phenomenon: Although increasing the $\theta$ value will accelerate the transformation from The Infiltrator to immunizer, so that the number of immunizers will increase rapidly in the early stage of transmission, but because a large number of potential communicators are transformed into immunizers in the early stage, the overall size of the immunizers will eventually decline. This shows that the high $\theta$ strategy can establish an effective immune barrier before rumors spread widely, thus significantly reducing the influence of rumors.

This discovery has important practical significance: by increasing the $\theta$ value, the spread of rumors can be more effectively curbed. This strategy of ”prevention is better than cure” is especially suitable for key scenes that need to quickly control the spread of rumors.

## Conclusion

In this paper, we develop a new model for rumor spreading in social networks based on current social situation and inspired by previous academic research. This model not only takes into account the real-world identities of individuals, but also extends the states of nodes on the basis of the original to have multiple states being accounted for. It provides a reliable basis for the development of effective rumor control strategies, and by simulating it on multiple real-world datasets, we draw the following important conclusions:

- Simulation results show that increasing the immunity rate can significantly reduce the number of infected nodes in the network, thus effectively slowing down the rumor spreading. Especially when there are more immunizers in the network, the propagation chain of rumor spreading will be cut off, which ultimately leads to the fading of rumors in the network. Therefore, enhancing the immunity of users in social networks, especially through education and information dissemination to improve the immunity rate, is an effective strategy to control the spread of rumors.
- By simulating on several real datasets, we find that the network’s topology has a significant impact on rumor propagation. For example, in networks with a high degree of centralization, rumors tend to spread rapidly through a few nodes, leading to large-scale infections. In contrast, in more decentralized networks, rumors spread relatively slowly and are less likely to cover the entire network. By optimizing the network structure and reducing the number of easy-to-spread nodes in the network, the scope and speed of rumor spreading can be effectively controlled.
- Compared with the traditional SIR model, this model is able to more accurately simulate the behaviors of different groups and individuals in a social network by introducing three identities (rumor publisher, rumor controller, and ordinary participant) and seven propagation states (active, inactive, the infiltrator, ignorant propagator, malicious propagator, wanderer, and immune). In particular, by refining the states of the nodes, we are able to observe the complex dynamics of the rumor propagation process, such as the transformation of rumor spreaders, the creation of immunizers, and key processes such as message blocking. This multi-dimensional modeling approach enhances the expressiveness of the model and provides higher prediction accuracy for practical applications.
- In the model, the interaction between rumor publishers and rumor controllers plays a crucial role in the final spreading effect of rumors. When the rumor publisher’s propagation rate exceeds the intervention ability of the rumor controller, the rumor tends to spread rapidly in the network. And when the immunity rate of rumor controllers is high, the chain of rumor spreading can be effectively blocked. Therefore, the game between rumor publishers and rumor controllers becomes an important dynamic feature in the model, further reflecting the complex social behavior in information dissemination.

Research and simulation analyses in this paper provide a powerful tool for understanding and controlling rumor propagation in social networks, and provide theoretical support for the solution of practical social problems. We believe that with the further research on social networks, dynamics-based communication models will become an important research direction to deal with the problems of disinformation and rumor spreading.

Research in this paper provides new ideas for modeling and controlling rumor spreading in social networks. However, there is potential for improvement for the current model, and future research could further extend the model to consider more complex network topologies, heterogeneity among nodes, and different types of interventions. For example, factors such as behavioral-based intervention strategies and the multi-level structure of social networks are worth exploring in depth. In addition, the parameter settings in the model (e.g. infection rate, recovery rate, etc.) can be further optimized to accommodate the transmission characteristics of different types of social networks.

## Data Availability

Data is provided within the manuscript or supplementary information files
